# Supplementary material for: Malaria and Helminthic Co-Infection during Pregnancy in Sub-Saharan Africa: A Systematic Review and Meta-Analysis
Source: Int J Environ Res Public Health. 2022 Apr 29;19(9):5444. doi: 10.3390/ijerph19095444 (PMC9101176; doi:10.3390/ijerph19095444)
Supplement: Supplementary file 1 [file ijerph-19-05444-s001.zip › supplemantary File S1 (search strategy).pdf]

EMBASE 155 articles retrieved

'coinfection'/exp OR **coinfection OR 'comorbidity'**/exp OR **comorbidity OR 'intestinal helminthiasis'**/exp OR **'intestinal helminthiasis' OR 'hookworm'**/exp OR **'hookworm' OR hookworm infection'**/exp OR **'hookworm infection' OR 'Ascaris lumbricoides'**/exp OR **'Ascaris lumbricoides' OR 'Ascariasis'**/exp OR **'Ascariasis' OR 'schistosomiasis'**/exp OR **'schistosomiasis' OR 'schistosoma mansoni'**/exp OR **'schistosoma mansoni' OR 'schistosoma hematobium'**/exp OR **'schistosoma hematobium' OR 'Trichuriasis'**/exp OR **'Trichuris trichuria' OR 'geohelminth'**/exp OR **geohelminth AND 'malaria'**/exp OR **malaria OR 'plasmodium vivax malaria'**/exp OR **'plasmodium vivax malaria' OR 'malaria falciparum'**/exp OR **'malaria falciparum' AND 'pregnant woman'**/exp OR **'pregnant woman' OR 'pregnant mother'** OR (**pregnant** AND (**'mother'**/exp OR **mother**))

CINAHL 159

"Co-infection*" OR "Comorbidity*" OR "Intestinal helminthiasis*" OR "soil transmitted helminthiases*" OR "geohelminth*" OR "Hookworm*" OR "Hookworm infection*" OR "Ascariasis*" OR "Ascaris lubricoides*" OR "Schistosomiasis*" OR "Schistosoma mansoni*" OR "Schistosoma hematobium*" OR "Trichuris trichuria*" OR "Trichuriasis*" AND "Plasmodium falciparum*" OR "Plasmodium vivax*" OR "Malaria*" AND "pregnant women*" OR "Pregnant mother*"

Google Scholar 481 (articles retrieved)

(Co-infection OR Comorbidity OR Intestinal helminthiasis OR Helminthic infection OR Soil-transmitted helminths OR Geohelminths OR Schistosomiasis OR Schistosoma hematobium OR Schistosoma mansoni OR Hookworm OR Hookworm infection OR Asacriasis OR Ascaris lumbricoides OR Trichuriasis OR Trichuris trichuria) AND (Malaria OR Plasmodium vivax OR Plasmodium falciparum OR Plasmodium malariae) AND (Pregnant Women OR Pregnant mother) AND (ANGOLA OR BENIN OR BOTSWANA OR BURKINA FASO OR BURUNDI OR CABO VERDE OR CAMEROON OR CENTRAL AFRICAN REPUBLIC OR CHAD OR COMOROS OR CONGO DEMOCRATIC REPUBLIC OR CONGO REPUBLIC OR COTE D'IVOIRE OR EQUATORIAL GUINEA OR ERITREA OR ESWATINI OR ETHIOPIA OR GABON OR GAMBIA OR GHANA OR GUINEA OR GUINEA-BISSAU OR KENYA OR LESOTHO OR LIBERIA OR MADAGASCAR OR MALAWI OR MALI OR MAURITANIA OR MAURITIUS OR MOZAMBIQUE OR NAMIBIA OR NIGER OR NIGERIA OR RWANDA OR SAO TOME AND PRINCIPE OR SENEGAL OR SEYCHELLES OR SIERRA LEONE OR SOMALIA OR SOUTH AFRICA OR SOUTH SUDAN OR SUDAN OR TANZANIA OR TOGO OR UGANDA OR ZAMBIA OR ZIMBABWE)

PubMed 499 (article retrieved)

(((((((((("Hookworm Infections"[Mesh]) OR ("Ascaris"[Mesh] OR "Ascaris lumbricoides"[Mesh] OR "Ascariasis"[Mesh])) OR ("Trichuris"[Mesh] OR "Trichuriasis"[Mesh])) OR ("Schistosoma"[Mesh] OR "Schistosoma mansoni"[Mesh] OR "Schistosoma haematobium"[Mesh] OR "Schistosomiasis mansoni"[Mesh] OR "Schistosomiasis haematobia"[Mesh])) OR "Intestinal helminthiasis" [Supplementary Concept]) OR "Anemia"[Mesh]) AND "Coinfection"[Mesh]) OR "Comorbidity"[Mesh]) OR ("Malaria"[Mesh] OR "Malaria, Vivax"[Mesh] OR "Malaria, Falciparum"[Mesh] OR "Acute malaria" [Supplementary Concept])) AND "Pregnancy"[Mesh]) OR "Pregnant Women"[Mesh] Filters: **Abstract, Free full text, Full text, Observational Study AND (Hookworm Infections*[tw] OR Soil-transmitted helminthiasis OR Ascaris*[tw] OR Ascaris lumbricoides*[tw] OR Ascariasis*[tw] OR Trichuris*[tw] OR Trichuriasis*[tw] OR Schistosoma*[tw] OR Schistosoma mansoni*[tw] OR Schistosoma haematobium*[tw] OR Schistosomiasis mansoni*[tw] OR Schistosomiasis haematobia*[tw] OR Intestinal helminthiasis*[tw] OR Anemia*[tw] AND Coinfection*[tw] OR Comorbidity*[tw] OR Malaria*[tw] OR Malaria, Vivax*[tw] OR Plasmodium vivax*[tw] OR Malaria, Falciparum*[tw] OR Plasmodium falciparum*[tw] OR Acute malaria*[tw] AND Pregnancy*tw] OR Pregnant Women*[tw] AND Angola*[tw] OR Benin*[tw] OR Botswana*[tw] or Burkina Faso*[tw] or Burundi*[tw] or Cabo Verde*[tw] or Cameroon*[tw] or Central African Republic*[tw] or Chad*[tw] or Comoros*[tw] or Congo, Democratic Republic of*[tw] or Congo, Republic of*[tw] or Cote d'Ivoire*[tw] or Equatorial Guinea*[tw] or Eritrea*[tw] or Eswatini*[tw] or Ethiopia*[tw] or Gabon*[tw] or Gambia*[tw] or Ghana*[tw] or Guinea*[tw] or Guinea-Bissau*[tw] or Kenya*[tw] or Lesotho*[tw] or Liberia*[tw] or Madagascar*[tw] or Malawi*[tw] or Mali*[tw] or Mauritania*[tw] or Mauritius*[tw] or Mozambique*[tw] or Namibia*[tw] or Niger*[tw] or Nigeria*[tw] or Rwanda*[tw] or Sao Tome and Principe*[tw] or Senegal*[tw] or Seychelles*[tw] or Sierra Leone*[tw] or Somalia*[tw] South Africa*[tw] South Sudan*[tw] or Sudan*[tw] or Tanzania*[tw] or Togo*[tw] or Uganda*[tw] or Zambia*[tw] or Zimbabwe*[tw])** Filters: **Abstract, Free full text, Full text, Observational Study**

Scopus 122 (articles retrieved)

(TITLE-ABS-KEY ("Coinfection") OR TITLE-ABS-KEY ("Comorbidity") OR TITLE-ABS-KEY ("Intestinal helminthiasis") OR TITLE-ABS-KEY ("Soil-transmitted helminths") OR TITLE-ABS-KEY ("Geohelminths") TITLE-ABS-KEY ("Schistosomiasis") OR TITLE-ABS-KEY ("Schistosoma hematobium") OR TITLE-ABS-KEY ("Schistosoma mansoni") OR TITLE-ABS-KEY ("Hookworm") OR TITLE-ABS-KEY ("Hookworm infection") OR TITLE-ABS-KEY ("Asacriasis") OR TITLE-ABS-KEY ("Ascaris lumbricoides") OR TITLE-ABS-KEY ("Trichuriasis ") OR TITLE-ABS-KEY ("Trichuris trichuria") AND TITLE-ABS-KEY ("Plasmodium vivax") OR TITLE-ABS-KEY ("Plasmodium falciparum") OR OR TITLE-ABS-

KEY ("Malaria") AND TITLE-ABS-KEY ("Pregnant Women") OR TITLE-ABS-KEY ("Pregnant mother")

Web of Science (253 articles retrieved)

("Co-infection*" OR "Comorbidity*" OR "Intestinal helminthiasis*" OR "Helminthic infection*" OR "Soil-transmitted helminths*" OR "Geohelminths*" OR "Schistosomiasis*" OR "Schistosoma hematobium*" OR "Schistosoma mansoni*" OR "Hookworm*" OR "Hookworm infection*" OR "Asacriasis*" OR "Ascaris lumbricoides*" OR "Trichuriasis*" OR "Trichuris trichuria*") AND ("Malaria*" OR "Plasmodium vivax*" OR "Plasmodium falciparum*" AND ("Pregnant Women*" OR "Pregnant mother*")
